# Changes in cigarette and e-cigarette use among US young adults from before to during the COVID-19 pandemic: News exposure and risk perceptions as potential predictors

**DOI:** 10.18332/tpc/148245

**Published:** 2022-05-06

**Authors:** Breesa Bennett, Katelyn F. Romm, Carla J. Berg

**Affiliations:** 1 Department of Epidemiology, Milken Institute School of Public Health, George Washington University, Washington, United States; 2 Department of Prevention and Community Health, Milken Institute School of Public Health, George Washington University, Washington, United States; 3 George Washington Cancer Center, George Washington University, Washington, United States

**Keywords:** COVID-19, tobacco, electronic cigarette, cigarette, young adults, cessation

## Abstract

**INTRODUCTION:**

COVID-19 impacted cigarette and e-cigarette use behaviors among some individuals. This study examined COVID-19 factors and prior substance use as predictors of cigarette and e-cigarette cessation and initiation among US young adults from before to during the COVID-19 pandemic.

**METHODS:**

We analyzed data from Wave 3 (Sept–Dec 2019) and Wave 5 (Sept–Dec 2020) of a 2-year, 5-wave longitudinal study of young adults across six US metropolitan areas. We examined COVID-19 news exposure, perceived smoking and e-cigarette use risk, and prior substance use, as predictors of cigarette and e-cigarette cessation and initiation, respectively.

**RESULTS:**

Of W3 cigarette users (n=516), 37.8% (n=195) quit cigarettes at W5; predictors of cessation included younger age, fewer days of W3 past-month cigarette use, and no W3 e-cigarette use. Of W3 e-cigarette users (n=687), 38.7% (n=266) quit e-cigarettes at W5; predictors included greater COVID-19 news exposure, fewer days of W3 past-month e-cigarette use, and no W3 cigarette use. Of W3 cigarette non-users (n=1693), 5.0% (n=85) initiated cigarettes at W5; predictors of initiation included younger age, lower perceived smoking risk, lifetime cigarette and e-cigarette use, and W3 e-cigarette use. Of W3 e-cigarette non-users (n=1522), 6.3% (n=96) initiated e-cigarettes at W5; predictors included younger age, less news exposure, lifetime cigarette and e-cigarette use, and W3 cigarette use.

**CONCLUSIONS:**

These findings underscore the need to address cigarette and e-cigarette co-use and related risk perceptions in prevention and cessation interventions.

## INTRODUCTION

COVID-19 was declared a global pandemic by the World Health Organization on 11 March 2020. Since the onset of COVID-19, public health efforts were put in place to mitigate the spread of the disease (e.g. social distancing, business closures), which resulted in a broad range of stressors – including job loss, social isolation, and distraction from normal life activities^1,2^. These negative mental health implications may also contribute to increased maladaptive coping-related substance use^1-3^. The potential for increased use of tobacco and/or nicotine products is particularly concerning, given some evidence that use of cigarettes^4-6^ and potentially e-cigarettes^7^ may increase the risk for contracting COVID-19 and/or be associated with worse COVID-19 disease outcomes (e.g. severity, mortality).

Young adults have among the highest rates of any tobacco use (18.2% past-month tobacco use)^8^, as well as cigarette and e-cigarette use (8.0% and 9.3%)^8^, and represent a substantial proportion of new users^9,10^. Given the potential risks of cigarette and e-cigarette use on COVID-19 health outcomes^4-6,11^ and some evidence that COVID-19 disproportionately impacted substance use among younger populations^12,13^, it is imperative to identify correlates of cessation and initiation of cigarette and e-cigarette use among young adults during the COVID-19 pandemic in order to inform related intervention efforts.

To examine predictors for changes in use of cigarettes and e-cigarettes among young adults during COVID-19, the current study draws from a socioecological perspective^14^, which underscores that individual behaviors result from dynamic interactions between individuals and their environment (e.g. physical, social, and political). Within this context, the COVID-19 pandemic introduced many impactful environmental stimuli (e.g. social distancing, economic devastation, massive media coverage) that potentially contributed to changes in cigarette or e-cigarette use, with some individuals increasing their use in response to stress and others reducing their use^3^. Considering individual/intrapersonal factors, greater perceived risk of COVID-19 (e.g. how much individuals perceived their use of e-cigarettes or cigarettes increased their risk of harm from COVID-19) has been shown to correlate with reducing and quitting cigarette and e-cigarette use^15-17^, aligning with risk perception theory^18^. One’s perceived risk is influenced by information and knowledge, for example, via media or news.^19^ Research suggests that exposure to COVID-19-related news is linked to perceived risk of COVID-19^20^ and to increased cigarette and e-cigarette use during the COVID-19 pandemic^21^.

Despite what literature exists, there is insufficient research assessing a broad range of factors associated with cigarette and e-cigarette use, initiation of these products, and longitudinal changes during the COVID-19 pandemic, particularly among young adults, who may be uniquely impacted by this period of societal stress^12^. Identifying a range of factors that are associated with initiation and cessation of cigarettes and e-cigarettes during COVID-19 is critical to informing prevention and intervention efforts aimed at curbing problematic patterns of their use during periods of societal stress. Thus, this study analyzed longitudinal data among young adults (aged 18–34 years) across six US metropolitan statistical areas (MSAs) and examined: 1) cessation of cigarette and e-cigarette use among current users from before (Fall 2019) to during (Fall 2020) the COVID-19 pandemic; 2) initiation of cigarette and e-cigarette use among non-users; and 3) COVID-19 related predictors of these outcomes. We hypothesized that individuals reporting greater COVID-19 news exposure and greater perceived smoking risk (e.g. greater health risks related to COVID-19 due to smoking) and e-cigarette use risk (e.g. greater health risks related to COVID-19 due to vaping) are: 1) more likely to report cigarette and e-cigarette cessation; and 2) less likely to initiate cigarettes and e-cigarettes.

## METHODS

### Study design

The current study analyzed survey data among young adults (aged 18–34 years) who participated in the Vape shop Advertising, Place characteristics and Effects Surveillance (VAPES) study, a 2-year, 5-wave longitudinal cohort study^22^. The VAPES study aims to examine the vape retail environment and its impact on substance use among young adults residing in six MSAs (Atlanta, Boston, Minneapolis, Oklahoma City, San Diego, and Seattle). Bi-annual survey assessments began in Fall 2018 (Sept–Dec) in the six MSAs selected for their state policy and retail markets for both tobacco and recreational marijuana^23^. Participants were surveyed every 6 months for 2 years (Sep–Dec; March–May), with mid-year assessments (in March–May) consisting of brief assessments of only key variables (e.g. cigarette and e-cigarette use). The VAPES study was approved by the Emory University Institutional Review Board.

### Participants and recruitment

Inclusion criteria for participation in the VAPES study were: 1) aged 18–34 years, 2) residing in one of the six MSAs, and 3) English speaking. Participants were recruited using advertisements on social media platforms (Reddit, Facebook) and sampled using purposive, quota-based sampling to obtain sufficient representation of cigarette and e-cigarette users (approximately 1/3 each), sexes (42.3% male), and racial/ethnic minorities (71.6% White, 11.4% Hispanic)^22^. Advertisements targeted potential participants by: 1) using keywords reflecting the eligibility criteria (e.g. young adult, MSA); 2) by presenting in groups appealing to young adults (e.g. followers or group members of pages related to sports, entertainment, or tobacco-related interests); and 3) by using images of young adults of diverse racial/ethnic backgrounds in various settings. Individuals that clicked on study advertisements were guided to a webpage that included a study description and consent form, and then screened for eligibility. Enrollment into subgroups was restricted in each MSA. Within 7 days of completing the baseline survey, participants were contacted via email and asked to confirm their participation in the study. The duration of recruitment ranged from 87–104 days across the six MSAs. In all, 10433 individuals clicked on the study advertisements, of which 9847 consented and 7096 were found eligible; 2751 (27.9%) did not advance to the baseline survey because they were either ineligible (n=1472) or excluded (n=1279); thus, 3006 participants completed the Wave 1 (W1) survey. Current analyses drew from data among participants who completed surveys at both Wave 3 (Sept–Dec 2019; n=2375; 79.0% retention) and Wave 5 (Sept–Dec 2020; n=2476; 82.4% retention). Participants received graduated compensation ($10 Amazon e-gift card at W1, $20 at W3, and $30 at W5) upon completion of each survey.

### Measures

*Primary outcomes: changes in substance use*

To operationalize both our outcomes and analytic samples, the following assessments (administered at W3 and W5) were used: ‘During the past 30 days, on how many days did you: a) smoke cigarettes (even a puff)?, b) use e-cigarettes (even a puff)?’. Participant use was dichotomized into any (>0 days of use) versus no use (0 days of use) for the respective products and at the respective waves.

Using this data, we operationalized the following outcomes among the distinct subsamples: 1) W5 cigarette cessation (i.e. no past-month cigarette use at W5) among W3 cigarette users (i.e. reported any past 30-day cigarette use at W3; n=516); 2) W5 e-cigarette cessation among W3 e-cigarette users (n=687); 3) W5 cigarette initiation (i.e. reported any past 30-day cigarette use at W5) among W3 cigarette non-users (i.e. no past-month cigarette use at W3; n=1693); and 4) W5 e-cigarette initiation among W3 e-cigarette non-users (n=1522).

These analyses included related variables as predictors. In models among W3 users, W3 level of use was included as a predictor, operationalized as a continuous variable of number of days used in the past 30 days for the respective product (cigarettes and e-cigarettes). Additionally, at W1 participants were asked to report whether they had used cigarettes or e-cigarette products in their lifetime (yes or no). Lifetime cigarette and e-cigarette use, respectively (yes vs no), were used as predictors in models among W3 non-users.

*Predictors: COVID-19 situational factors*

At W5, participants were asked whether they had been ‘laid off’ due to COVID-19 (yes vs no). COVID-19 stress was assessed by asking participants their agreement to the following: 1) ‘the COVID-19 pandemic has been (or was) extremely stressful for me’; 2) ‘the COVID-19 pandemic distracted me from doing other important things in my life’; and 3) the COVID-19 pandemic made me feel very lonely and distant from people’ (1=strongly disagree to 5=strongly agree). A mean score of these 3 items was computed to create an overall COVID-19 stress variable, with higher scores indicating greater stress (Cronbach’s α=0.80). To assess ‘perceived smoking and e-cigarette use risk’, participants rated their agreement to the following items: ‘Smoking puts people at greater health risks related to COVID-19’ and ‘Vaping puts people at greater health risks related to COVID-19’ (1=strongly disagree to 5=strongly agree). COVID-19 news exposure was assessed by asking participants: ‘How closely do you follow COVID-19 news, either in the newspapers or on television, radio, or the Internet?’ (1=not at all closely to 4=very closely).

*Covariates: sociodemographics*

Sociodemographics assessed included: age, sex, sexual orientation, race, ethnicity, education level, employment status, relationship status, and whether they had children (aged <18 years) in their home.

### Data analysis

Descriptive analyses and bivariate analyses (i.e. chi-squared and t-tests) were used to characterize the sample and subsamples of W3 cigarette and e-cigarette users and non-users who used or did not use at W5. Hierarchical multivariable logistic regression analyses were conducted among the 4 subgroups (as indicated above) to predict cigarette cessation, e-cigarette cessation, cigarette initiation, and e-cigarette initiation from W3 to W5. We entered our primary predictors of interest – COVID-19 situational factors – in the first block. Additional variables entered in the second block were selected based on bivariate results; specifically, we chose to include age and past cigarette and e-cigarette use. We excluded some sociodemographics in multivariable models due to multicollinearity (e.g. age correlated with education, employment, relationship status, and children in the home, so only age was included). Analyses were conducted using SPSS v26, and alpha was set at 0.05.

## RESULTS

Bivariate comparisons among past 30-day cigarette users and e-cigarette users at W3 in relation to use status at W5 are shown in Table 1, and among past 30-day cigarette non-users and e-cigarette non-users at W3 in relation to use status at W5 are shown in Table 2. Multivariable regression analyses are presented in Table 3.

**Table 1:** Bivariate comparisons among past 30-day cigarette users (N=516) and e-cigarette users (N=687) at W3 in relation to use status at W5

*Variables*	*W5 Cigarette Cessation*			*W5 E-cigarette Cessation*		
	*Total*	*Yes*	*No*	*Total*	*Yes*	*No*
	*n (%)*	*n (%)*	*n (%)*	*n (%)*	*n (%)*	*n (%)*
**Total**	516 (100)	195 (37.8)	321 (62.2)	687 (100)	266 (38.7)	421 (61.3)
**MSA**						
Atlanta	87 (16.9)	41 (21.0)	46 (14.3)	123 (17.9)	50 (18.8)	73 (17.3)
Boston	98 (19.0)	29 (14.9)	69 (21.5)	126 (18.3)	58 (21.8)	68 (16.2)
Minneapolis	100 (19.4)	42 (21.5)	58 (18.1)	123 (17.9)	39 (14.7)	84 (20.0)
Oklahoma City	54 (10.5)	18 (9.2)	36 (11.2)	72 (10.5)	24 (9.0)	48 (11.4)
San Diego	84 (16.3)	37 (19.0)	47 (14.6)	120 (17.5)	45 (16.9)	75 (17.8)
Seattle	93 (18.0)	28 (14.4)	65 (20.2)	123 (17.9)	50 (18.8)	73 (17.3)
**Age** (years), Mean ± SD	25.43 ± 4.76	**24.49 ± 4.54**	**26.00 ± 4.81**	24.18 ± 4.72	23.99 ± 4.56	24.30 ± 4.83
**Sex**						
Male	252 (48.8)	89 (45.6)	163 (50.8)	321 (46.7)	115 (43.2)	206 (48.9)
Female	245 (47.5)	95 (48.7)	150 (46.7)	349 (50.8)	145 (54.5)	204 (48.5)
**Sexual minority**	183 (35.5)	81 (41.5)	102 (31.8)	237 (34.5)	97 (36.5)	140 (33.3)
**Race**						
White	371 (71.9)	137 (70.3)	234 (72.9)	498 (72.5)	193 (72.6)	305 (72.4)
Black	31 (6.0)	12 (6.2)	19 (5.9)	30 (4.4)	14 (5.3)	16 (3.8)
Asian	47 (9.1)	20 (10.3)	27 (8.4)	68 (9.9)	25 (9.4)	43 (10.2)
Other	67 (13.0)	26 (13.3)	41 (12.8)	91 (13.2)	34 (12.8)	57 (13.5)
**Hispanic**	81 (15.7)	**16 (8.2)**	**65 (20.2)**	89 (13.0)	35 (13.2)	54 (12.8)
**Education** (≥Bachelor's degree)	329 (63.8)	133 (68.2)	196 (61.1)	442 (64.3)	**188 (70.7)**	**254 (60.3)**
**Employment**						
Student	112 (21.7)	51 (26.2)	61 (19.0)	158 (23.0)	71 (26.7)	87 (20.7)
Unemployed	64 (12.4)	23 (11.8)	41 (12.8)	60 (8.7)	**31 (11.7)**	**29 (6.9)**
Full-time	213 (41.3)	67 (34.4)	146 (45.5)	258 (37.6)	**87 (32.7)**	**171 (40.6)**
Part-time	127 (24.6)	54 (27.7)	73 (22.7)	211 (30.7)	77 (28.9)	134 (31.8)
**Relationship status**						
Married/living with partner	216 (41.9)	73 (37.4)	143 (44.5)	255 (37.1)	97 (36.5)	158 (37.5)
Single/other	300 (58.1)	122 (62.6)	178 (55.5)	432 (62.9)	169 (63.5)	263 (62.5)
**Children in the home**	145 (28.1)	**40 (20.5)**	**105 (32.7)**	147 (21.4)	53 (19.9)	94 (22.3)
**COVID-19 factors**						
Laid off from job	98 (19.0)	38 (19.5)	60 (18.7)	130 (18.9)	47 (17.7)	83 (19.7)
COVID stress, Mean ± SD	3.88 ± 1.02	**4.06 ± 1.00**	**3.77 ± 1.02**	3.85 ± 1.07	3.91 ± 1.11	3.81 ± 1.04
Perceived smoking risk, Mean ± SD	4.02 ± 1.06	**4.26 ± 0.95**	**3.88 ± 1.10**	4.10 ± 1.08	**4.25 ± 1.10**	**4.01 ± 1.06**
Perceived e-cigarette use risk, Mean ± SD	3.79 ± 1.11	**3.99 ± 1.05**	**3.67 ± 1.13**	3.79 ± 1.16	**4.01 ± 1.15**	**3.66 ± 1.14**
COVID news exposure, Mean ± SD	3.13 ± 0.84	**3.27 ± 0.78**	**3.05 ± 0.86**	3.17 ± 0.81	**3.28 ± 0.78**	**3.10 ± 0.82**
**W3 current (past 30-day) use**						
Cigarettes, Mean ± SD	11.33 ± 11.12	**6.75 ± 9.15**	**14.11 ± 11.31**	-	-	-
E-cigarettes, Mean ± SD	-	-	-	14.72 ± 12.12	**8.73 ± 10.27**	**18.52 ± 11.67**
Cigarettes	-	-	-	336 (48.9)	**109 (41.0)**	**227 (67.6)**
E-cigarettes	336 (65.1)	120 (61.5)	216 (67.3)	-	-	-

MSA: US metropolitan statistical area. Bolded values denote statistical significance.

**Table 2:** Bivariate comparisons among past 30-day cigarette non-users (N=1693) and e-cigarette non-users (N=1522) at W3 in relation to use status at W5

*Variables*	*W5 Cigarette Initiation*			*W5 E-cigarette Initiation*		
	*Total*	*Yes*	*No*	*Total*	*Yes*	*No*
	*n (%)*	*n (%)*	*n (%)*	*n (%)*	*n (%)*	*n (%)*
**Total**	1693 (100)	85 (5.0)	1608 (95.0)	1522 (100)	96 (6.3)	1426 (93.7)
**MSA**						
Atlanta	376 (22.2)	10 (11.8)	366 (22.8)	340 (22.3)	**10 (10.4)**	**330 (23.1)**
Boston	369 (21.8)	21 (24.7)	348 (21.6)	341 (22.4)	21 (21.9)	320 (22.4)
Minneapolis	287 (17.0)	22 (25.9)	265 (16.5)	264 (17.3)	20 (20.8)	244 (17.1)
Oklahoma City	170 (10.0)	8 (9.4)	162 (10.1)	152 (10.0)	**18 (18.8)**	**134 (9.4)**
San Diego	265 (15.7)	12 (14.1)	253 (15.7)	229 (15.0)	15 (15.6)	214 (15.0)
Seattle	226 (13.3)	12 (14.1)	214 (13.3)	196 (12.9)	12 (12.5)	184 (12.9)
**Age** (years), Mean ± SD	24.47 ± 4.62	**22.80 ± 4.32**	**24.56 ± 4.62**	24.92 ± 4.63	**23.88 ± 5.07**	**24.99 ± 4.59**
**Sex**						
Male	665 (39.3)	44 (51.8)	621 (38.6)	596 (39.2)	44 (45.8)	552 (38.7)
Female	991 (58.5)	39 (45.9)	952 (59.2)	887 (58.3)	50 (52.1)	837 (58.7)
**Sexual minority**	499 (29.5)	25 (29.4)	474 (29.5)	445 (29.2)	28 (29.2)	417 (29.2)
**Race**						
White	1204 (71.1)	62 (72.9)	1142 (71.0)	1077 (70.8)	73 (76.0)	1004 (70.4)
Black	86 (5.1)	4 (4.7)	82 (5.1)	87 (5.7)	5 (5.2)	82 (5.8)
Asian	237 (14.0)	10 (11.8)	227 (14.1)	216 (14.2)	13 (13.5)	203 (14.2)
Other	166 (9.8)	9 (10.6)	157 (9.8)	142 (9.3)	5 (5.2)	137 (9.6)
**Hispanic**	163 (9.6)	9 (10.6)	154 (9.6)	155 (10.2)	8 (8.3)	147 (10.3)
**Education** (≥Bachelor's degree)	1361 (80.4)	**57 (67.1)**	**1304 (81.1)**	1248 (82.0)	**69 (71.9)**	**1179 (82.7)**
**Employment**						
Student	507 (29.9)	25 (29.4)	482 (30.0)	461 (30.3)	28 (29.2)	433 (30.4)
Unemployed	113 (6.7)	6 (7.1)	107 (6.7)	117 (7.7)	7 (7.3)	110 (7.7)
Full-time	687 (40.6)	**23 (27.1)**	**664 (41.3)**	642 (42.2)	34 (35.4)	608 (42.6)
Part-time	386 (22.8)	**31 (36.5)**	**355 (22.1)**	302 (19.8)	27 (28.1)	275 (19.3)
**Relationship status**						
Married/living with partner	605 (35.7)	24 (28.2)	581 (36.1)	566 (37.2)	37 (38.5)	529 (37.1)
Single/Other	1088 (64.3)	61 (71.8)	1027 (63.9)	956 (62.8)	59 (61.5)	897 (62.9)
**Children in the home**	282 (16.7)	16 (18.8)	266 (16.5)	280 (18.4)	**26 (27.1)**	**254 (17.8)**
**COVID-19 factors**						
Laid off from job	200 (11.8)	14 (16.5)	186 (11.6)	168 (11.0)	13 (13.5)	155 (10.9)
COVID stress, Mean ± SD	3.95 ± 0.98	3.88 ± 1.06	3.95 ± 0.98	3.97 ± 0.95	3.97 ± 0.95	3.97 ± 0.95
Perceived smoking risk, Mean ± SD	4.48 ± 0.88	**4.06 ± 1.18**	**4.50 ± 0.85**	4.50 ± 0.85	**4.04 ± 1.07**	**4.53 ± 0.82**
Perceived e-cigarette use risk, Mean ± SD	4.23 ± 0.98	**3.86 ± 1.24**	**4.25 ± 0.96**	4.28 ± 0.92	**3.80 ± 1.08**	**4.31 ± 0.90**
COVID news exposure, Mean ± SD	3.26 ± 0.72	**3.06 ± 0.92**	**3.28 ± 0.71**	3.26 ± 0.72	**2.89 ± 0.86**	**3.29 ± 0.71**
**Ever/lifetime use**						
Cigarettes	612 (36.1)	**69 (81.2)**	**543 (33.8)**	566 (37.2)	**78 (81.3)**	**488 (34.2)**
E-cigarettes	818 (48.4)	**76 (89.4)**	**742 (46.2)**	614 (40.4)	**86 (89.6)**	**528 (37.1)**

MSA: US metropolitan statistical area. Bolded values denote statistical significance.

**Table 3:** Characteristics associated with W5 cigarette cessation among W3 cigarette users, W5 e-cigarette cessation among W3 e-cigarette users, W5 cigarette initiation among W3 cigarette non-users, and W5 e-cigarette initiation among W3 e-cigarette non-users

*Variable*	*Quit cigarettes W3-W5*			*Quit e-cigarettes W3-W5*			*Initiated cigarettes W3-W5*			*Initiated e-cigarettes W3-W5*		
	*AOR*	*95% CI*	*p*	*AOR*	*95% CI*	*p*	*AOR*	*95% CI*	*^p^*	*AOR*	*95% CI*	*^p^*
**Block 1 – COVID-19 factors**												
Perceived smoking risk	1.37	1.05–1.79	**0.022**	0.96	0.76–1.21	0.743	0.68	0.47–0.99	**0.042**	0.80	0.57–1.13	0.205
Perceived e-cigarette use risk	1.03	0.81–1.32	0.792	1.33	1.07–1.65	**0.010**	0.99	0.69–1.43	0.951	0.78	0.56–1.09	0.151
Level of COVID-19 news exposure	1.30	1.04–1.64	**0.023**	1.31	1.07–1.61	**0.009**	0.74	0.56–0.99	**0.041**	0.56	0.43–0.73	**<0.001**
Nagelkerke R^2^		0.056			0.045			0.037			0.076	
**Block 2 – COVID-19 factors**												
Perceived smoking risk	1.33	1.00–1.77	0.051	1.00	0.78–1.29	0.987	0.68	0.47–0.99	**0.043**	0.86	0.60–1.23	0.398
Perceived e-cigarette use risk	1.03	0.79–1.33	0.857	1.19	0.94–1.51	0.153	1.07	0.74–1.54	0.733	0.78	0.55–1.10	0.154
Level of COVID-19 news exposure	1.26	0.99–1.61	0.066	1.25	1.00–1.56	**0.046**	0.87	0.64–1.17	0.352	0.63	0.48–0.84	**0.002**
**Sociodemographics and Prior use**												
Age	0.95	0.91–0.99	**0.012**	1.02	0.98–1.06	0.411	0.88	0.82–0.93	**<0.001**	0.89	0.84–0.94	**<0.001**
Number of days of cigarette use	0.94	0.92–0.96	**<0.001**	-	-	-	-	-	-	-	-	-
Number of days of e-cigarette use	-	-	-	0.93	0.92–0.95	**<0.001**	-	-	-	-	-	-
W3 current cigarette use (Ref: no)	-	-	-	0.56	0.39–0.80	**0.001**	-	-	-	7.57	3.82–14.99	**<0.001**
W3 current e-cigarette use (Ref: no)	0.62	0.41–0.94	**0.024**	-	-	-	4.45	1.98–10.00	**<0.001**	-	-	-
Ever cigarette use (Ref: no)	-	-	-	-	-	-	6.62	3.48–12.58	**<0.001**	3.47	1.80–6.68	**<0.001**
Ever e-cigarette use (Ref: no)	-	-	-	-	-	-	2.41	1.06–5.50	**0.036**	6.33	3.06–13.10	**<0.001**
Nagelkerke R^2^		0.204			0.237			0.240			0.305	

AOR: adjusted odds ratio. Bolded values denote statistical significance.

### Cigarette cessation

Among W3 cigarette users (n=516), 37.8% (n=195) quit cigarettes at W5 (Table 1). Those who quit using at W5: were significantly younger, more likely sexual minority, non-Hispanic, and not living with children; reported significantly greater COVID-19 related stress, perceived smoking risk, perceived e-cigarette use risk, and COVID-19 news exposure; and reported significantly fewer days of W3 cigarette use (Mean ± SD: 6.75 ± 9.15) compared to W3 users who did not quit at W5 (14.11 _±_ 11.31, p<0.001). Multivariable regression analyses (Table 3) indicated that greater perceived smoking risks and more COVID-19 news exposure predicted W5 cigarette cessation among W3 cigarette users (Block 1). When including sociodemographics and prior cigarette and e-cigarette use (Block 2), predictors included younger age (adjusted odds ratio, AOR=0.95; 95% CI: 0.91–0.99), fewer days of W3 cigarette use (AOR=0.94; 95% CI: 0.92–0.96), and no W3 e-cigarette use (AOR=0.62; 95% CI: 0.41–0.94).

### E-cigarette cessation

Among W3 e-cigarette users (n=687), 38.7% (n=266) quit e-cigarettes at W5 (Table 1). Those who quit at W5: were significantly more likely to have a Bachelor’s degree or higher, more likely to be unemployed, and less likely to be employed full-time; reported significantly greater perceived smoking risk, perceived e-cigarette use risk, and COVID-19 news exposure; significantly less likely to report W3 past 30-day cigarette use; and reported significantly fewer days of W3 e-cigarette use (8.73 ± 10.27) compared to W3 users who did not quit at W5 (18.52 ± 11.67, p<0.001). In multivariable regression analyses (Table 3), predictors of W5 e-cigarette cessation among W3 e-cigarette users included greater perceived risks of e-cigarette use and more COVID-19 news exposure (Block 1). When including sociodemographics and prior cigarette and e-cigarette use (Block 2), predictors included greater COVID-19 news exposure (AOR=1.25; 95% CI: 1.00–1.56), fewer days of W3 e-cigarette use (AOR=0.93; 95% CI: 0.92–0.95), and no W3 cigarette use (AOR=0.56; 95% CI: 0.39–0.80).

### Cigarette initiation

Among W3 cigarette non-users (n=1693), 5.0% (n=85) initiated cigarettes at W5 (Table 2). Those who initiated cigarette use at W5: were significantly younger, less likely to have a Bachelor’s degree or higher, more likely to be employed part-time and less likely to be employed full-time; were significantly more likely to report lifetime cigarette and e-cigarette use; and reported significantly lower perceived smoking risk, perceived e-cigarette use risk, and COVID-19 news exposure. Multivariable analyses (Table 3) indicated that predictors of W5 cigarette initiation among W3 cigarette non-users included lower perceived smoking risks and less COVID-19 news exposure (Block 1). When including sociodemographics and prior cigarette and e-cigarette use (Block 2), predictors included younger age (AOR=0.88; 95% CI: 0.82–0.93), lower perceived smoking risk (AOR=0.68; 95% CI: 0.47–0.99), lifetime cigarette (AOR=6.62; 95% CI: 3.48–12.58), and e-cigarette use (AOR=2.41; 95% CI: 1.06–5.50), and W3 e-cigarette use (AOR=4.45; 95% CI: 1.98–10.00).

### E-cigarette initiation

Among W3 e-cigarette users (n=1522), 6.3% (n=96) initiated e-cigarettes at W5 (Table 2). W3 e-cigarette non-users who initiated e-cigarette use at W5: were significantly younger, more likely to reside in Oklahoma City and less likely to reside in Atlanta; were significantly less likely to have a Bachelor’s degree or higher, and more likely living with children; reported significantly lower perceived smoking risk, perceived e-cigarette use risk, and COVID-19 news exposure; and were significantly more likely lifetime cigarette and e-cigarette users. Findings from multivariable regression analyses (Table 3) indicated that, among W3 e-cigarette non-users, less COVID-19 news exposure predicted W5 e-cigarette initiation (Block 1). When including sociodemographics and prior cigarette and e-cigarette use (Block 2), predictors included younger age (AOR=0.89; 95% CI: (0.84–0.94), less COVID-19 news exposure (AOR=0.63: 95% CI: 0.48–0.84), lifetime cigarette (AOR=3.47; 95% CI: 1.80–6.68) and e-cigarette use (AOR=6.33; 95% CI: 3.06–13.10), and W3 cigarette use (AOR=7.57; 95% CI: 3.82–14.99).

## DISCUSSION

This study examined change in cigarette and e-cigarette use status among young adults during the COVID-19 pandemic. Among young adults who used these substances during Fall 2019 (W3), nearly 40% quit use of cigarettes or e-cigarettes by Fall 2020 (W5), 37.8% and 38.7%, respectively. Other research has found that cigarette and e-cigarette quit rates during the pandemic were 26% and 41% among US adults aged ≥18 years^24^. Quit rates among young adults in particular – outside the COVID-19 pandemic period – have been shown to be 21.6% and 55.6% for cigarettes and e-cigarettes, respectively^25^.

Roughly 5% to 6% of young adults who did not use cigarettes or e-cigarettes at W3 initiated use of these products at W5, respectively. Other research has found that cigarette and e-cigarette initiation rates during the pandemic were 6.9% and 4.4% among US adults aged ≥18 years^26^. Initiation rates among younger adults – outside the COVID-19 pandemic period – have been shown to be 3.6% and 4.5% for cigarettes and e-cigarettes, respectively^27^.

Consistent with a socioecological perspective^14^, changes in cigarette and e-cigarette use were predicted by individual factors, specifically past substance use and perceived risk, and contextual factors, specifically COVID-19 news exposure. With regard to individual factors, not surprisingly, common predictors across all outcomes were prior cigarette and e-cigarette use behaviors, as a wealth of research would suggest^25,27-31^. For example, fewer days of W3 past-month cigarette use and no W3 e-cigarette use, predicted cigarette cessation; fewer days of W3 past-month e-cigarette use and no W3 cigarette use, predicted e-cigarette cessation; lifetime cigarette and e-cigarette use and W3 e-cigarette use, predicted cigarette initiation; and lifetime cigarette and e-cigarette use and W3 cigarette use, predicted e-cigarette initiation. These findings also underscore the interconnectivity of these behaviors. Those who quit cigarettes or e-cigarettes were less likely to use the alternative product at W3, which provides further evidence of the particular risks of cigarette and e-cigarette co-use with regard to chronic use and development of addiction^29,30,32,33^. Those who initiated use of cigarettes or e-cigarettes were more likely to have used the other product at W3, providing further support for similar underlying mechanisms contributing to their use and for these use behaviors to cluster among young adults over time^27,29-31,33^.

Among our primary predictors of interest, novel findings were noted. For example, in models controlling for sociodemographics and prior substance use, lower perceived smoking risk predicted cigarette initiation only; perceived e-cigarette use risk did not significantly predict any outcome. In the models not controlling for sociodemographics or prior substance use, greater perceived risk of cigarette and e-cigarette use predicted cigarette and e-cigarette cessation, respectively. However, lower perceived risk of e-cigarette use was not associated with e-cigarette initiation. These findings coincide with risk perception theory^18^ and prior evidence of these associations^15-17^. However, these findings are novel as they indicate that perceived risk of using one product does not impact the use of the other, and that perceived risk of e-cigarette use did not predict e-cigarette initiation. Thus, young adults’ differing perceptions of the products – and the fact that e-cigarette initiation is likely driven by other factors, such as social influences^32,34^ – point to the importance of better understanding the underlying mechanisms that contribute to different product use, cigarette and e-cigarette co-use, and cessation.

Also noteworthy is that greater COVID-19 news exposure predicted e-cigarette cessation and less news exposure predicted e-cigarette initiation. While news exposure did not predict cigarette use outcomes when controlling for sociodemographics and prior substance use, the models not controlling for these factors showed similar associations for cigarette use outcomes as for e-cigarette use outcomes. These findings indicate that COVID-19 related news exposure had the potential to change behavior, in this case cigarette and e-cigarette use, adding to the limited existing research^21^.

Interestingly, being younger predicted both cigarette cessation and e-cigarette initiation and cigarette initiation. These findings are difficult to interpret within the context of prior research. For example, previous research has shown that young adults aged 25–34 years are at greater risk of using cigarettes, while young adults aged 18–24 years are at particular risk of using e-cigarettes^10^. Thus, findings documented here may be related to cohort effects and the natural timing of when young adults may be initiating versus quitting different types of products – or potentially using one product (e-cigarettes) to quit the other (cigarettes)^35^.

### Strengths and limitations

This study benefits from a longitudinal design and data collection during critical periods before and during the pandemic, a large sample size, and sufficient subgroups that made these analyses possible, and the inclusion of novel assessments across a broad range of factors. These strengths are critical given the limitations of the existing literature; for example, a large proportion of studies on related topics used cross-sectional data obtained during the COVID-19 pandemic^3,15,16,21^, which has been shown to yield notably different findings from longitudinal prospective data collections^36^.

However, the study has some limitations, including limited generalizability to other young adults in the included MSAs or across the US. Cigarette and e-cigarette use rates should not be interpreted as prevalence rates, as our sampling design oversampled current cigarette and e-cigarette users to achieve a sample with approximately a third being cigarette and e-cigarette users, respectively. This study utilized self-reported data, and self-reported cigarette and e-cigarette use data in particular may be subject to recall and social desirability bias. Finally, although guided by a socioecological perspective^14^, the measures included in these analyses were not exhaustive of all potential factors influencing cigarette and e-cigarette use outcomes among young adults but rather focused on a selected set of novel factors. This decision, in part, was due to the fact that, despite the sample sizes were sufficient for each of the sets of analyses, the number of people reporting specific outcomes (e.g. cigarette and e-cigarette initiation) was small, thus requiring us to restrict the number of predictors considered. Other factors – for example, parental or peer substance use influences^34^ – should also be examined in future research.

## CONCLUSIONS

In this sample of young adults, nearly 40% of cigarette or e-cigarette users quit using the respective product from before to during the COVID-19 pandemic. However, 5% and 6% initiated use of cigarettes and e-cigarettes, respectively. Importantly, prior cigarette and e-cigarette use predicted not only their respective future use but also use of the other product. Interestingly, however, perceived risk of each product distinctly predicted future use of the respective product. Moreover, COVID-19 news exposure demonstrated a potential protective role in these outcomes. These findings point to the need to consider information sources – particularly the media – and perceptions of these products, as well as individual use profiles, in developing interventions to reduce cigarette and e-cigarette use in young adults, particularly during societal stressors, like the COVID-19 pandemic.

## Data Availability

The data supporting this research are available from the authors on reasonable request.
